# T71. CHANGE AND STABILITY IN COGNITIVE TRAJECTORIES FROM CHILDHOOD TO LATE ADOLESCENCE IN YOUNG OFFSPRING AT GENETIC RISK OF SCHIZOPHRENIA AND MOOD DISORDER: IMPLICATIONS FOR THE RISK STATUS

**DOI:** 10.1093/schbul/sby016.347

**Published:** 2018-04-01

**Authors:** Elsa Gilbert, Thomas Paccalet, Valérie Jomphe, Daphné Lussier, Michel Maziade

**Affiliations:** 1 Université Laval; 2 Université de Québec; 3 CERVO Brain Research Centre; 4 Université Laval, CERVO Brain Research Centre

## Abstract

**Background:**

Cognitive impairments are a core feature of schizophrenia (SZ).1 The few existing retrospective or prospective population-based studies indicate that patients who develop psychoses have cognitive impairments in childhood and adolescence.2,3 Offspring at high genetic risk also present neurocognitive impairments before the age of disease incidence.4,5 The form of the childhood cognitive trajectory may be a predictor of transition to illness but more data are needed on the early cognitive trajectories in children at genetic risk to inform about the most sensitive periods in risk progression to psychosis and to orient interventions.6 The objective was to investigate the cognitive trajectories in children born to a parent affected by a SZ or BP from early childhood to late adolescence, in terms of changes in cognitive 4 domains known to be impaired in major psychosis. A special attention was given to the timing of changes in childhood, and their association with well documented clinical risk indicators.

**Methods:**

The sample consisted of 79 offspring (age from 6 to 21) born to parents affected by SZ or BP from our multi-affected kindreds of Eastern Quebec. Our cognitive battery covered: episodic memory, working memory, speed of processing and executive functioning evaluated at two-time points (mean duration between assessments +/- 6y). A Cognitive domain was considered impaired when mean performance was below -1 SD. We had measurements of established childhood risk indicators [4]: psychotic-like experiences, non-psychotic DSM diagnoses and social functioning (GAF).

**Results:**

Three distinct developmental trajectories were identified according to the progression in number of impaired cognitive domains from baseline to follow-up: i) A “steady” trajectory with stable and intact performances across all cognitive domains (n=52; 66%); ii) a “deteriorating” trajectory with an accumulation of cognitive impairments (n=18; 23%) and; iii) an “improving” trajectory with a diminishing number of cognitive impairments at follow-up (n=9; 11%). IQ and neuropsychological performances were similar at baseline between the “deteriorating” and “improving” trajectories (p=.4), while the steady group performed best. The 3 subgroups were comparable in terms of the parent diagnosis and offspring gender. Regarding clinical risk indicators, the deteriorating subgroup presented a worsening of social functioning between the two-time points (-7 GAF points vs -0.8 for steady, +0.56 for improving) and a higher rate of childhood non-psychotic DSM diagnosis (p≤.01). Importantly, we observed striking differences in cognitive trajectories among siblings suggesting that change or stability go beyond the heritability of cognitive capacities.

**Discussion:**

Our results suggest three types of cognitive developmental trajectories among offspring of parents affected by SZ or BP. The progressive deteriorating trajectory was associated with an aggregation of other clinical risk indicators shown to predict transition.4 Cognitive deterioration was slightly more frequent in childhood and pre-adolescence than in late adolescence which has implications for the timing of detection, the need of care, the type of longitudinal surveillance and the design of future prevention research.6

**References:**

1. Keefe & Kahn, JAMA Psychiatry, 2017

2. Meier et al., Am J Psychiatry, 2014

3. MacCabe et al., JAMA Psychiatry 2013

4. Paccalet et al., Schizophr Res, 2016

5. Maziade et al., Schizophr Bull, 2011

6. Maziade, N Eng J Med, 2017

